# Risk factors for porcine reproductive and respiratory syndrome virus infection and resulting challenges for effective disease surveillance

**DOI:** 10.1186/1746-6148-8-184

**Published:** 2012-10-04

**Authors:** Martina Velasova, Pablo Alarcon, Susanna Williamson, Barbara Wieland

**Affiliations:** 1 The Royal Veterinary College, Hawkshead Lane, North Mymms, Hatfield, Hertfordshire, United Kingdom; 2 Animal Health and Veterinary Laboratories Agency, Rougham Hill, Bury St Edmunds, Suffolk, United Kingdom

## Abstract

**Background:**

This study aimed to identify risk factors for active porcine reproductive and respiratory syndrome virus (PRRSV) infection at farm level and to assess the probability of an infected farm being detected through passive disease surveillance in England. Data were obtained from a cross-sectional study on 147 farrow-to-finish farms conducted from April 2008 – April 2009. The risk factors for active PRRSV infection were identified using multivariable logistic regression analysis. The surveillance system was evaluated using a stochastic scenario tree model.

**Results:**

Evidence of PRRSV circulation was confirmed on 35.1% (95%CI: 26.8-43.4) of farms in the cross sectional study, with a higher proportion of infected farms in areas with high pig density (more than 15000 pigs within 10 km radius from the farm). Farms were more likely to have active PRRSV infection if they used the live virus vaccine-Porcilis PRRS (OR=7.5, 95%CI: 2.5-22.8), were located in high pig density areas (OR=2.9, 95%CI: 1.0-8.3) or had dead pigs collected (OR=5.6, 95%CI: 1.7-18.3). Farms that weaned pigs at 28 days of age or later had lower odds of being PRRSV positive compared to those weaning at 21-27 days (OR=0.2, 95%CI: 0.1-0.7). The probability of detecting an infected farm through passive surveillance for disease was low (mode=0.074, 5th and 95th percentiles: 0.067; 0.083 respectively). In particular farms which used live virus vaccine had lower probabilities for detection compared to those which did not.

**Conclusions:**

Risk factors identified highlight the importance of biosecurity measures for the incursion of PRRSV infection. The results further indicate that a combined approach of surveillance for infection and disease diagnosis is needed to assist effective control and/or elimination of PRRSV from the pig population.

## Background

In England and worldwide, porcine reproductive and respiratory syndrome (PRRS) is considered to be one of the most important diseases affecting pigs [1]. This is mainly due to its impact on production, especially as the virus is putatively immunosuppressive and concurrent diseases are common [2-4]. The resulting economic impact of PRRS on pig production can be significant, especially if it occurs in herds or regions with no previous history of infection. In individual herds, direct costs relate to production losses, increased mortality and reproductive failure. Indirect costs are mainly associated with treatment, disease control, pig disposal costs and disruption to breeding programmes. In the USA, it was estimated that the cost of PRRS to the pig industry may be as high as $641 million per year [5]. In the UK, the cost of PRRS in growing and fishing pigs on non-vaccinated farms during the acute phase was estimated to be as high as £52 180 for a 500 sow herd and as high as £40 000 in a breeding herd of similar size. During the chronic phase, the estimated cost of PRRS per year was £34 823 in vaccinated growing and finishing pigs and overall up to £93 590 for the breeding herd in the year following a breakdown [6].

The disease was first reported in the USA in 1987 and by 1990 it had spread throughout North America (NA) [7]. Almost simultaneously, but independently from NA, the disease emerged in Europe. The first European country to report PRRS was Germany in 1990 [8] followed by the Netherlands [9,10], Belgium [11] and Spain [12]. The PRRS virus (PRRSV) was first isolated in the Netherlands in 1991 (Lelystad isolate) [9] and shortly afterwards in the USA (VR-2332 isolate) [13]. Both isolates now define the two main genotypes of the PRRSV, genotype 1 (European) and genotype 2 (North American) [14,15]. These genotypes cause similar clinical signs but differ significantly genetically and antigenically [16-18]. Within the European genotype, distinct clusters of genetic subtypes have been identified [19,20].

As an RNA virus, PRRSV is prone to mutation and, over time, the diversity of the virus of both genotypes has increased [21-23]. The increasing genetic diversity may result in strains which break through the efficacy of current PRRS vaccines and undermine PRRS control based on the use of vaccination only. This makes it all the more important to understand the extent of disease and infection in vaccinated and unvaccinated units in order to design effective control strategies.

In Britain, the first clinical cases were confirmed in 1991 [24]. Since then, the virus has spread and is now considered endemic in the UK. Based on data collected in 2003/2004, Evans et al. (2008) estimated 40% of seropositive non-vaccinated and 26% of vaccinated farms in the UK. To date, there is only evidence of circulation of European genotype of PRRSV (AHVLA unpublished data). Clinical manifestation on infected farms is influenced by a number of inter-acting factors including the naivety of the pigs to the infecting strain, vaccination, management practices, environmental stressors and presence of other pathogens, such as *Mycoplasma hyopneumoniae and Actinobacillus* pleuropneumoniae [25]. In endemically infected herds, disease can be mild or even subclinical compared to herds with recent infection in naïve pigs [26,27]. As one of the control measures, vaccination for PRRS using either the European genotype live vaccine (Porcilis PRRS) or killed vaccines (Progressis and Ingelvac PRRS) has been implemented. Based on field observations, herds that vaccinate are likely to be those that are infected or are at risk of becoming infected.

Surveillance is an important tool to generate information on detection and distribution of disease or infection in the animal population [28,29]. Since there is no active surveillance for PRRSV undertaken in the UK, diagnosis of disease outbreaks due to PRRS is achieved through passive disease surveillance. Passive surveillance is defined as the reporting of clinical suspect cases to the health authorities [30]. In the UK, it is achieved through the submission and testing of diseased pigs, tissues and blood samples from diseased pigs and is undertaken by the Regional Laboratories (RLs) of the Animal Health and Veterinary Laboratories Agency (AHVLA) and, in Scotland, the Scottish Agricultural College. PRRSV detected then usually represent submissions from either PRRS outbreaks due to new breakdowns of negative herds or uncontrolled PRRSV in positive herds. In recent years, there has been a slight decrease in submissions, but the proportion of submissions diagnosed with PRRS has increased from 2% to 10% [31].

Identification of risk factors is important to identify and implement adequate control measures and to design cost effective surveillance strategies. Various studies have been carried out to investigate risk factors for PRRSV infection at herd level in England and elsewhere. Increased herd size, distance to the nearest pig herd [3], pig and herd density, purchase of semen [32], increased purchase of gilts and boars, and total confinement housing [33] were found to be associated with increased risk of PRRSV infection. In recent years, the situation on many farms has changed. Despite the implementation of various control measures, including the vaccination, changes to management practices or breed genetics, PRRS continues to be a major problem for many pig producers. The wide distribution of PRRSV and the risk of reintroduction into herds after eradication [14], undermines control efforts.

This study aimed to evaluate the prevalence of PRRSV infection in a study population of English pig herds, to identify risk factors for active PRRSV infection at herd level and to assess the probability that an infected herd will be detected through passive disease surveillance.

## Results

### Herd prevalence of PRRSV infection

In total, 147 farms were recruited in this study between April 2008 and April 2009. The median herd size was 300 sows, range 16 to 2,000 sows. Single site farrow to finish production was observed on 84 farms (57.1%) and on multiple sites on 63 farms (42.9%). All-indoor type of production was the most common type, and 26.7% of farms kept either all or most of their pigs outdoors. Geographically, all regions in England were represented, but two regions (North Yorkshire and East Anglia) accounted for more than half of recruited farms. Geographic distribution of studied farms corresponded to the pig population density in England. Common biosecurity measures in place were no purchase of breeding stock (43.4%), buying breeding stock one to six times per year (18.9%), having dead pigs collected (69.1%), using on-farm incinerator (30.9%), protective clothing for staff and visitors (76.6%), and requirements of visitors to be pig-free (80.4%) with a median of two days (minimum of one and maximum seven days). Further, 49.3% of farmers reported the weaning age of piglets between 21 to 27 days (median 26 days) and 50.7% of farmers between 28 and 45 days (median 28 days). The latter farms were slightly smaller herds (median= 250, min=39, max=950) compared to those weaning at 21-27 days (median=377.5, min=130, max=2000).

Sixty-three farms (42.8%) reported use of PRRSV vaccination, with 52 (82.5%) using live and 11 (17.5%) killed vaccine. On 16 seropositive farms that vaccinated, circulation of the virus was not confirmed by PCR and therefore were classified as dubious and excluded from further analysis. In total, 46/131 farms (35.1%, 95%CI: 26.8-43.4) tested positive for PRRSV and of those, 28 used live vaccine, two used killed vaccine and the remaining 16 used no vaccination. The proportion of positive farms on non-vaccinated and vaccinated farms with live and killed vaccine is summarised in Table 1. Of the vaccinated farms, four farms were classified as PRRSV positive based on only one seropositive and one virus positive sample. On two of these farms, a finisher pig tested positive to antibodies and virus, as did a grower pig on another of these farms. On the remaining farm, a finisher pig tested positive to antibodies and a grower pig tested positive to virus. On non-vaccinated farms, five farms were classified as PRRSV positive based on only one grower pig testing positive. The number of PRRSV positive farms according to different age groups (grower, finisher) is summarised in Table 2. In 62.2% of PRRSV positive farms the farmers believed their farms to be PRRSV-infected, while 84.8% of the PRRSV negative farms were considered PRRSV-free by the farmer. More than 85% of positive farms were found in high pig density areas with fewer farms in the Midlands, one in South West and none in the South East of England.

**Table 1:** Proportion of PRRSV positive farms amongst all farms and according to different vaccination status, 16 farms were classified as dubious and were excluded from further analysis

	**N**	**Number of positive (%)**	**95 % Confidence Interval**
All farms	131	46 (35.1)	26.8-43.4
Non-vaccinated	84	16 (19.5)	10.4-27.6
Vaccinated:	47	30 (63.8)	49.5-78.1
Live vaccine	39	28 (71.8)	57.0-86.5
Killed vaccine	8	2 (25.0)	13.7-63.7

**Table 2:** Number of farms that were classified as PRRSV positive according to different age groups

	**Vaccinated (n=30)**		**Non-vaccinated (n=16)**
	**ELISA**	**PCR**	**ELISA**
	**N (%)**	**N (%)**	**N (%)**
-growers	6 (20.0)	19 (63.3)	5 (31.2)
-finishers	14 (46.7)	8 (26.7)	3 (18.8)
-growers + finishers	10 (33.3)	3 (10.0)	8 (50.0)

Further evidence of exposure to at least one other pathogen tested for (APP, swine influenza, PCV2) on PRRSV positive farms was common; 53.3% of PRRSV positive farms also tested positive for H1N2, 31.1% for avian-like H1N1, 84.8% for APP and 89.1% for PCV2. Ten of 46 PRRS positive farms tested positive for both avian-like H1N1 and H1N2 and 34 farms for both APP and PCV2. On PRRSV negative farms, 40.0% tested positive for H1N2, 12.9% for avian-like H1N1, 65.9% for APP and 89.4% for PCV2. Five of 85 PRRSV negative farms tested positive for both avian-like H1N1 and H1N2 and 51 for both APP and PCV2.

### Risk factor analysis

On three farms insufficient data were obtained and they were therefore excluded from the risk factor analysis. Table 3 summarizes the exposure variables associated with PRRSV positive farms in the univariable analysis (p ≤ 0.20).

**Table 3:** Summary of exposure variables associated with PRRSV status in the univariable analysis (p≤0.2)

**Variable name**	**Value**	**Number of PRRSV positive (%)**	**Number of PRRSV negative (%)**	**OR**	**95% CI**	**P-value**
Herd size	<250	14 (31.1)	38 (48.1)	1.0		
(Number of sows)	≥250	31(68.9)	41(51.9)	2.0	0.9-4.4	0.06
Farm type	outdoor	8 (17.8)	29 (34.5)	1.0		
	indoor	37 (82.2)	55 (65.5)	2.4	1.0-5.9	0.05
PRRS vaccine	none	16 (34.8)	68 (80.0)	1.0		
	killed	2 (4.3)	6 (7.1)	1.4	0.2-7.6	
	live	28 (60.9)	11 (12.9)	10.8	4.4-26.2	<0.01
APP ELISA	negative	7 (15.2)	29 (34.1)	1.0		
	positive	39 (84.8)	56 (65.9)	2.9	1.1-7.2	0.02
Avian-like H1N1	negative	31 (68.9)	74 (87.1)	1.0		
	positive	14 (31.1)	11 (12.9)	3.0	1.2-7.4	0.01
Age at weaning in days	21-27	30 (65.2)	33 (40.7)	1.0		
	≥28	16 (34.8)	48 (59.3)	0.3	0.1-0.7	<0.01
Disposal of dead pigs	incineration	6 (13.0)	33 (42.9)	1.0		
	collection	40 (87.0)	44 (57.1)	5.0	1.8-13.1	<0.01
Frequency of live	never	14 (31.1)	22 (28.2)	1.0		
animals	1-6/year	11 (24.4)	45 (57.7)	0.3	0.1-0.9	
	>6/year	20 (44.5)	11 (14.1)	2.8	1.0-7.7	<0.01
Pig density*	<15000	20 (43.5)	68 (81.0)	1.0		
	≥15000	26 (56.5)	16 (19.0)	5.5	2.4-12.2	<0.01
Other production species	no	34 (73.9)	44 (53.0)	1.0		
	yes	12 (26.1)	39 (47.0)	0.4	0.1-0.8	0.02
Number of farm	1-2	5 (10.8)	28 (35.0)	1.0		
workers	3	16 (34.8)	20 (25.0)	4.4	1.4-14.2	
	4	14 (30.4)	12 (15.0)	6.5	1.9-22.2	
	>4	11 (23.9)	20 (25.0)	3.1	0.9-10.2	0.01
Use of straw yards	no	16 (38.1)	43 (53.1)	1.0		
	yes	26 (61.9)	38 (46.9)	1.8	0.8-3.9	0.11
Ventilation weaners	natural	22 (47.8)	54 (64.3)	1.0		
	artificial	14 (30.4)	20 (23.8)	1.7	0.7-3.9	
	both	10 (21.7)	10 (11.9)	2.4	0.9-6.7	0.15
Ventilation growers	natural	26 (56.5)	61 (73.5)	1.0		
	artificial	7 (15.2)	9 (10.8)	1.8	0.6-5.4	
	both	13 (28.3)	13 (15.7)	2.3	0.9-5.7	0.13
Ventilation finishers	natural	22 (51.1)	60 (72.3)	1.0		
	artificial	14 (32.6)	12 (14.5)	3.1	1.2-7.9	
	both	7 (16.3)	11 (13.2)	1.7	0.6-5.0	0.04
Ventilation lactating	natural	16 (34.8)	43 (51.8)	1.0		
sows	artificial	21 (45.6)	28 (33.7)	2.0	0.9-4.5	
	both	9 (19.6)	12 (14.5)	2.0	0.7-5.6	0.17
Lighting weaners	natural	15 (32.6)	41 (48.8)	1.0		
	artificial	13 (28.3)	17 (20.2)	2.1	0.8-5.3	
	both	18 (39.1)	26 (31.0)	1.9	0.8-4.4	0.19
Lighting lactating	natural	9 (19.6)	30 (36.1)	1.0		
sows	artificial	21 (45.6)	22 (26.5)	3.2	1.2-8.3	
	both	16 (34.8)	31 (37.4)	1.7	0.6-4.5	0.04
Presence of cattle	no	40 (87.0)	64 (77.1)	1.0		
	yes	6 (13.0)	19 (22.9)	0.5	0.2-1.3	0.18
Presence of poultry	no	41 (89.1)	64 (77.1)	1.0		
	yes	5 (10.9)	19 (22.9)	0.4	0.1-1.1	0.10
Large white in breed	0	2 (4.4)	13 (16.3)	1.0		
composition in %	1-25	36 (80.0)	55 (68.7)	4.2	0.9-19.9	
	>25	7 (15.6)	12 (15.0)	3.8	0.6-21.9	0.11

*total number of pigs within 10km radius from the farm, OR: odds ratio, CI: confidence interval.

After controlling for the effect of herd size and production type (outdoor/indoor), four risk factors for PRRSV infection were identified in the multivariable analysis (Table 4). Farms using PRRSV live vaccine had higher odds of being PRRSV positive compared to non-vaccinated farms. Further, farms where dead pigs are collected were more likely to test PRRSV positive compared to those using on-farm incinerators, farms in high pig density areas had also higher odds to test positive for PRRSV. Age at weaning ≥ 28 days was identified as a protective factor compared with early weaning (21-27 days). The adjusted Hosmer-Lemeshow goodness of fit test indicated no problems with the fitted model (p=0.16).

**Table 4:** **Risk factors for PRRSV infection identified in multivariable logistic regression analysis, model adjusted for herd size and production type (outdoor/indoor), N=117, R**^**2**^**=0.35**

**Variable name**	**Value**	**N(%)**	**OR**	**95% CI**	p-value*
PRRS vaccine	none	74 (63.3)	1.0		
	killed	6 (5.1)	0.5	0.1-5.6	0.55
	live	37 (31.6)	7.5	2.5-22.8	<0.01
Dead pigs disposal	incineration	38 (32.5)	1.0		
	collection	79 (67.5)	5.6	1.7-18.3	<0.01
Pig density (in 10 km radius)	<15000	80 (68.4)	1.0		
	≥15000	37 (31.6)	2.9	1.0-8.3	0.04
Age at weaning in days	21-27	57 (48.7)	1.0		
	≥28	60 (51.3)	0.2	0.1-0.7	<0.01

*Wald test p-value, OR: odds ratio, CI: confidence interval.

### Probability of an infected farm being detected through disease surveillance

Farms located in high pig density areas using live vaccine had the highest effective probability of infection (EPI) compared to other risk strata. Estimated numbers of herds per risk stratum and corresponding EPIs are summarized in Table 5. The probability of infected farms being detected through passive pig disease surveillance in different risk strata is also shown in Table 5. When all 2962 registered pig farms were considered, the mode probability of detection was 7.4%. When stratified by risk strata, farms located in high and low density areas which did not use live vaccine had higher mode for the probability of being detected compared to those who used live vaccine.

**Table 5:** The probability that farms infected with PRRSV will be detected through passive disease surveillance assuming 35% herd prevalence considering all farms and farms in individual risk strata

	**Probability of detection of infected farm**			**Percentiles**	
	**n**	EPI*	**Mode**	**5**^**th**^	**95**^**th**^
All farms	2962	-	0.074	0.067	0.083
HDA* + live vaccine	303	1.000	0.069	0.054	0.084
HDA + no live vaccine	538	0.307	0.091	0.074	0.106
LDA* + live vaccine	765	0.425	0.068	0.053	0.084
LDA + no live vaccine	1356	0.113	0.089	0.074	0.106

The probabilities are shown as proportions.

*HDA=high pig density area, LDA=low pig density area, EPI=effective probability of infection.

The results from the sensitivity analysis suggested that probability of an infected pig showing clinical signs was the most important input parameter. When doubling the proportion of animals showing clinical signs, the mode for probability of detection of infected farm changed from 0.074 to 0.112 (5th- 95th percentiles: 0.101-0.121) and when halving it changed to 0.037 (5th - 95th percentiles: 0.032-0.044).

## Discussion

This study aimed to improve understanding of the epidemiology of PRRSV infection by estimating the herd prevalence of PRRSV in England and by identifying possible risk factors for active PRRSV infection. Despite some limitations, the findings from this study agree with previous findings of risk factors studies and provide some new evidence of the factors involved in the epidemiology of PRRSV infection. The number of recruited farms representing 65 000 sows (approx. 14% of the total sow population in the UK in 2008), their geographic distribution and type of farms, suggest a good representation of English farrow-to-finish farms in this study. Further, when compared with a previous study carried out in England by Evans et al. (2008); farms of similar size were recruited.

The estimated herd prevalence of active PRRSV infection (35.1%) indicates continuous virus circulation or recent virus introduction on a number of farms despite efforts to control the infection. This finding is similar to that of a previous study carried out in England in 2003-2004, where the evidence of virus presence and transmission on the farm was seen in 25 non-vaccinated herds (32.8%) with seropositive young stock [3]. Including the adult pigs in the sample of the same study, in total 41 of 76 non-vaccinated herds (53.9%) were seropositive. A similar result of 56% PRRSV seropositive herds was obtained through a diagnostic service offered to veterinary surgeons, carried out in Great Britain in 2001-2003 [34].

Sample size, sensitivity and specificity of the ELISA and PCR test and the possibility of selection bias needs to be considered when interpreting the results of this study. The initial recruitment of farms was conducted through the PCV2 vaccination program which could have resulted in the recruitment of a higher proportion of farms with more health problems than average and thus slightly overestimated herd prevalence of active PRRSV infection in England. However, to minimize the impact of this selection bias, 20% of farms were recruited through pig veterinarians and these farms were believed to have very few if any health problems. This study only included farrow-to-finish farms, and therefore results cannot be extrapolated to other types of farms. On the farms were growing pigs are reared on multiple sites, close contacts between the sites were observed and since these farms are continuously occupied by growing pigs of different ages, they are more likely to favour endemic PRRSV infection compared to breeding farms where young susceptible pigs are removed, or compared to single age all-in, all-out rearing units. The contact structure of the herds has previously been found to be an important factor for maintaining the virus within a farm [35].

Classification of a farm as PRRSV positive was based on growing and finishing pigs only. Therefore no vaccinated animals were included in the case definition. Testing pigs on seropositive vaccinating farms by PCR confirmed active circulation of PRRSV in these herds and reduced misclassification bias. Classification as PRRSV positive based on one positive ELISA only was made on five non-vaccinated farms. On these farms, it was a grower pig testing positive in the ELISA test, with finishers testing seronegative. Antibodies in these younger pigs but not in older finishers could indicate a longer than normal persistence of maternal antibodies [36,37] rather than exposure to active infection and this may have resulted in misclassification of PRRS status on these five farms. Correlation of antibodies and virus (Table 2) on vaccinated farms is in agreement with the findings reported previously where number of seropositive animals increases with age and prevalence of virus decreases with age [38]. Further, sensitivity (Se) and specificity (Sp) of the diagnostic tests used needs to be considered. The Sp of the Biobest in-house ELISA was validated as being greater than 95% and should result in no more than 5% of false positives on non-vaccinated farms. Less than 100% Se could result in some infected farms being missed; however, as results were interpreted at herd-level, the overall Se improved. The lack of PCR testing on all farms could also have resulted in misclassification bias. Ideally, a case definition would be based on the same laboratory test for all farms and would include use of both ELISA and PCR for evaluation of active PRRSV infection [38]. Further, the detection of the virus on all sampled farms would be more indicative of recent virus infection than the use of antibody ELISA test. Due to financial constraints this was not feasible here. However having ELISA positive animals among both growers and finishers on non-vaccinated farms was considered sufficient evidence of recent virus circulation on these farms. This assumption is in agreement with findings from a previous study, where seropositive unvaccinated young stock was considered to indicate virus presence on that farm [3].

Taking into account manufacturer information on sensitivity (Se) and specificity (Sp) of the ELISA test (both > 95%) a farm would be correctly classified if the herd prevalence was 60% or higher (Free Calc, Version 2), which is realistic considering estimates of within herd prevalence found by others [3,34,38]. On the other hand, the detection of virus on vaccinated farms may be limited by the sample size used as a lower within herd prevalence is expected due to vaccination. Variability in virus prevalence between eight to 30% in growing pigs reported in the study by Duinhof et al, (2011), could have resulted in our sample size being insufficient to detect the virus and thus obtaining false negative results. Accordingly, vaccinated farms which were seropositive but virus negative were classified as dubious and were excluded from further analysis.

This study identified a high proportion (> 85%) of positive farms in North Yorkshire and East Anglia. This finding is similar to a previous report from England with regional variation of seropositive farms apparent in Yorkshire (82%) and East Anglia (56%) [34] . Both regions contain significant proportions of the pig herds in England and together account for more than half of the overall pig population. There are pig dense areas within both regions with pig units in close geographical proximity to each other. There may also be close relationships between these units sharing the same supply chains for pigs, feed and pig vehicles, which increases the risk of transmission of infection between units [23,27,35,39]. In addition, aerosol transmission of the virus from infected herds over several kilometres, 4.7 km [40] and up to 9.1 km [41] under certain meteorological conditions, has also been demonstrated.

Using a multivariable logistic regression model, several risk factors for PRRSV infection were identified. A number of exposure variables related to management practices and biosecurity such as: type of ventilation for lactating sows and weaners; type of lighting for lactating sows; presence of cattle and poultry; and number of farm workers was found to be associated with PRRS farm status in the univariable analysis (Table 3). However, due to strong collinearity (p<0.01) with other exposures they were not retained for further analysis.

Farms using live virus vaccine were more likely to be PRRSV positive compared to non-vaccinated farms. This is not unexpected since farms which vaccinate are those which are likely to have experienced PRRS problems in the past and indeed, the majority of farmers of infected farms indicated that they believe to be infected. Also PRRS vaccination does not provide sterile immunity or prevent infection, but prevents clinical disease and reduces virus replication [42,43], thus detection of PRRSV in nearly all vaccinating herds was not surprising. Based on PCR results alone it was not possible to determine whether the virus detected was the live vaccine or a field strain, in particular considering that studies elsewhere have detected vaccine virus in both vaccinated and non-vaccinated pigs [44]. This was observed mostly with vaccine virus related to the North American (NA) genotype [44-47]. On the other hand, only limited transmission of the European genotype vaccine virus could be demonstrated [44,48]. Considering the circulation of only European genotype of PRRSV in the UK and the limited transmission of European genotype vaccine virus, the presence of virus in growers and finishers was considered more likely to reflect a field virus challenge, although virus sequencing would be necessary to confirm this. Results should therefore be interpreted with caution as occasional vaccine virus circulation may have resulted in misclassification of herds.

Overall, the risk factors identified were not surprising and support known routes of virus transmission [23,27,35,39]. They further agree with previous findings from England and elsewhere where herd size and pig density were found to be associated with increased risk of PRRSV infection and thus highlight the importance of biosecurity measures in preventing incursion of PRRSV [3,32,33]. Two identified risk factors for infection, collection of dead pigs and increased pig density, suggest that direct transmission from the pigs, or indirect from vehicles and people involved in their handling is an important factor for infection. Farms weaning piglets at the age of ≥ 28 days (28-45 days of age) had lower odds of being PRRSV positive compared to those weaning slightly earlier (21-27 days of age). Further analysis, after accounting for production type, found an association between weaning age and herd size. Farms weaning at the age of 28 days and later were more likely to be smaller herds (OR=0.3, 95%CI: 0.1-0.6, p<0.01). Previous studies suggested that in smaller herds, PRRSV is more likely to fade out [3,35,39]. This could be used to explain the association of the weaning age with farms’ PRRSV status observed in this study.

Two risk factors, pig density and vaccination with live virus, were used for the development of the stochastic model to evaluate the detection of an infected farm through passive pig disease surveillance. Farmers’ perception as to whether they believed their farm was infected by PRRSV was used to estimate the proportion of farms showing clinical signs suspicious of PRRS. The use of this information could have resulted in overestimation of this parameter since clinical signs were not seen by farmers on all of these farms. However, as clinical signs were identified to be the most sensitive input parameter, more robust collection of the data such as mortality records, growth performance and respiratory signs suspicious of PRRS in growing pigs and reproduction performance in breeding stock should be performed in the future. Furthermore, the AHVLA pig disease surveillance system supports diagnosis of any pig disease and the presence of clinical signs is an important factor prompting submission of material for diagnosis, thus presence of clinical signs was found to be most important for detecting PRRSV infected farms. Lack of clinical signs and poor recognition of clinical signs [26] could contribute to PRRSV infection remaining undetected on farms with active infection. Instituting PRRS-specific control measures could be delayed on such farms and they may therefore remain a source of infection to other pig units. The lower probability of detection of infection in farms using live vaccination compared to those without live vaccination (Table 5) is likely to reflect the reduction of clinical signs due to vaccination [34,43]. While non-significant in the multivariable analysis, the presence of concurrent pathogens on PRRSV positive farms detected in the univariable analysis is important as it affects the morbidity and severity of clinical signs such as respiratory disease and ill thrift [26,49,50]. Submissions to AHVLA under passive surveillance diagnosed as PRRS tend to be from severe or unusual disease outbreaks or from herds breaking through natural or vaccine immunity [23]. Such submissions provide important material for monitoring the PRRSV diversity and it has been confirmed that the virus has become more genetically diverse since the introduction into the UK [23].

Improved knowledge of herd prevalence and geographical distribution of PRRSV in England as well as an understanding of risk factors is important for more effective control of PRRSV. Given the estimated herd prevalence of PRRSV and the results from the scenario tree model, surveillance needs to be enhanced to support control of PRRSV. Knowledge of the risk factors for PRRSV infection could be used to develop more cost effective targeted surveillance which would include diagnosis of infection as well as disease and thus help current control programmes in England.

## Conclusions

This study estimated the prevalence of active PRRSV infection in English pig herds and found an association between high pig density areas and higher prevalence of infection, suggesting that current control measures are not effective at eliminating and maintaining freedom from PRRSV. The risk factors identified highlight the importance of good biosecurity practices in control of PRRSV. The findings of the scenario tree modelling also highlight the need for a combined approach of surveillance for PRRSV infection as well as disease to allow effective control and prevention of losses due to PRRS. Successful control of PRRS will contribute to the improvement of the overall pig health and positively impact on the competitiveness of British pork.

## Methods

### Study population and data collection

The data used for this study were collected between April 2008 and April 2009 in the context of a cross-sectional study on post-weaning multi-systemic wasting syndrome (PMWS) in England. Farms were recruited through the BPEX (the British pig levy payer association) PCV2 vaccination scheme, and high health farms through private veterinary practitioners. Inclusion criteria restricted recruitment to farrow-to-finish farms with growing pigs reared on single or multiple sites. All farms were visited prior to implementation of the PCV2 vaccination. During a one-day visit to each farm, through interview with the farmers and through on-farm assessment, data were collected on general farm characteristics, management practices, environmental conditions, production parameters, health status, vaccination programmes, animal welfare, genetics, breeding performance, and biosecurity measures. The opinion of the farmers as to whether they believed their farm was affected by PRRS at that time was also recorded.

From each farm, 12 blood samples were collected (six 11 to 14-week-old growers and six finishers15-weeks-old or more). All samples were tested for antibodies to PRRSV (Biobest-in house ELISA, Biobest Laboratories Ltd., UK). On farms where PRRS vaccination was in use and which were found to be PRRSV seropositive, available samples were also tested for virus using a PRRSV specific real time PCR (RT-PCR, AnDiaTec AcuPig, Biobest Laboratories Ltd., UK) in order to confirm circulation of virus. Seronegative farms were considered as negative and were not further tested by RT-PCR. A sample of 12 pigs/herd (6 growers and 6 finishers) was sufficient to detect at least one PRRSV positive animal with 95% confidence if the within herd sero- or virus prevalence was 22% or higher (Win Episcope 2.0). This is true for the PCR test where sensitivity (Se) and specificity (Sp) of up to 100% can be assumed. In addition to PRRS, the samples were also tested for the presence of *Actinobacillus pleuropneumoniae* APP (APP, Swinecheck^R^ ELISA specific for serotypes 3, 6 and 8, Biovet, Saint-Hyacinthe, Quebec, Canada) and swine influenza virus (avian-like H1N1, H1N2 and H3N2 strains) using haemagglutination inhibition test. Porcine circovirus type 2 (PCV2) was detected using a real time PCR protocol described elsewhere [51]. Written informed consent was obtained from each participating farm. All blood testing was considered as clinical-diagnostic care and results were fed back to farmers and their veterinarians as soon as available. Therefore no further formal approval of the Home Office was required.

### PRRS case definition

A positive farm was one with evidence of active or recent, rather than historical, PRRSV infection. Unvaccinated farms were classified as positive if at least one grower or finisher tested positive for PRRSV antibody. Vaccinated farms were classified as positive if at least one grower or finisher tested positive in ELISA test and presence of virus was confirmed in at least one grower or finisher through RT-PCR. PRRS vaccination on farms in this study was mainly used in breeding pigs only; therefore a PCR positive grower or finisher pig was taken to indicate active circulation of the virus on the farm.

### Statistical analysis

Data collected during farm visits were entered into a Microsoft Access 2007 database and transferred to Stata 11.2 (StataCorp, College station, Texas) for further analysis. Farm information and data on biologically plausible risk factors collected were divided into five variable groups (see Table 6 for details).

**Table 6:** Exposure variables included in the risk factor analysis for PRRS infection

**Variable group**	**Variable description**
1. General farm information	Geographic location (by region)
	Number of sites (one/multiple)
	Herd size (measured by number of sows)
	Pig density (total number of pigs within 10 km radius from the farm)
	Farm type: outdoor/ indoor
2. Herd health	*Farmers perception:*
	Herd vaccination program (PPV, PRRS)
	Type of PRRS vaccine used (none/killed virus /live virus)
	*Results from serology and PCR:*
	Serological results: APP, SI (avian-like H1N1, H1N2, H3N2)
	PCR results: PCV2 (PCR)
3. General management practices	*Herd environment:*
	Ventilation type (natural/artificial/both)
	Lightening (natural/artificial/both)
	Presence of slurry system (yes/no)
	Use of straw yards at any stage of the production (yes/no)
	Presence of other animal species on the farm (yes/no)
	Weaners stocking density/pen
	Growers stocking density/pen
	Finishers stocking density/pen
	*Herd management:*
	Number of movements between weaning and finishing
	Mixing of pigs at any stage of the production (yes/no)
	All in all out system at any stage of the production (yes/no)
	Use of sick/hospital pens on farm (yes/no)
	Routine cross-fostering performed (yes/no)
4. Genetics	*Breed composition* (percentage of breeds in the average finishing pig):
	Large White (LW)
	Landrace (LD)
	Pietrain (P)
	Duroc (D)
	Hampshire (H)
	Miesham (M)
5. Biosecurity	*Possible route of disease introduction through people:*
	Number of people working on the farm
	Average number of visitors/month
	Number of days pig free
	Use of protective clothes (yes/no)
	Use of boot dips (yes/no)
	Presence of fences around the farm (yes/no)
	Allowed parking on the farm (yes/no)
	*Possible route of disease introduction through animals:*
	Purchase of boars (yes/no)
	Purchase of gilts (yes/no)
	Purchase of semen (yes/no)
	Disposal of dead pigs (collection/incineration/other)

Deviations from the normal assumption for continuous variables were investigated using histograms and the D'Agostino test for skewness and kurtosis. Appropriate log transformations or categorizations, using quartiles or cut offs published elsewhere [3], were performed accordingly. A new variable was created for the frequency of bringing breeding pigs onto the farm if the farmer was purchasing gilts and/or boars. This was done to minimize the correlation between variables purchasing gilts and purchasing boars and, thus, to avoid their potential exclusion from further analysis of the risk factors. Data relating to the pig density (total number of pigs within a 10km radius of the farm) were taken from the 2004 UK agricultural census [52]. Univariable analysis of each exposure variable with the binary outcome was performed using Chi-squared test and Chi-squared test for trend for categorical variables and univariable logistic regression for continuous exposure variables. The association of any variable to the outcome was tested at a relaxed significance level alpha = 20%. When significant the variables were considered for multivariable regression modeling. Within each exposure group, exposure variables retained after univariable analysis were checked for collinearity using Spearman correlation coefficient for continuous variables and Chi-squared test for categorical variables. Two variables were considered to be collinear if a significance level alpha of 1% and a correlation coefficient > 0.7 was obtained. If collinearity occurred, the variable with the stronger association with the outcome or better biological plausibility for PRRS was retained and included in the multivariable logistic regression model.

Confounding was assessed by adding variables into the model starting with exposure variables with the strongest association from univariable analysis with the outcome. Each time an exposure variable was added, confounding was examined and a likelihood ratio (LR) test was done to assess whether the variable should remain in the model. Two a priori confounders were considered (herd size and production type: outdoor/indoor) and forced into the model at all times. Ordered categorical exposure variables were checked for linear trend using LR test.

Finally, a backward stepwise variable selection process based on a significance level alpha of 5% was used. All possible two-way interactions were assessed and retained if they improved model fit as determined by LR test (significance level alpha of 5%) and were biologically plausible. Overall model fit was assessed using adjusted Hosmer-Lemeshow goodness of fit test [53].

### Evaluation of detection of PRRSV through disease surveillance

**Figure 1:**
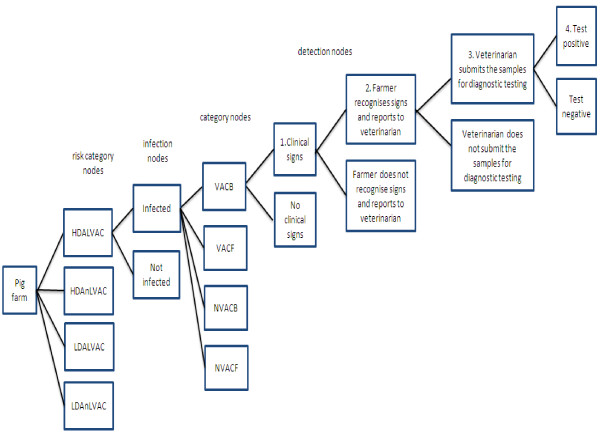
**Scenario tree. **Scenario tree for pig disease surveillance system through which PRRS is diagnosed on pig farms in England. See Table 7 for an explanation of the abbreviations used for individual nodes in this figure. Only the branches for the HDALVAC risk node are shown, the same branches were used for the other risk nodes (HDAnVAC, LDALVAC, LDAnLVAC).

The evaluation of passive disease surveillance system component (SSC) was based on stochastic scenario tree approach which was originally developed to demonstrate freedom from disease using multiple complex data sources [54]. In this method, the chain of events from an animal being infected to being detected is used to estimate the probability, i.e. sensitivity of detecting at least one infected animal/herd at a predefined prevalence [54]. As PRRSV is considered endemic in the UK, for the current study the approach described by Martin et al. (2007) was adapted in order to estimate the probability that an infected farm is identified through the existing passive disease surveillance in different risk strata. Two risk category nodes were used, PRRS live vaccination and pig density (high: more than 15000 pigs within 10 km radius from the farm and low: less than 15000 pigs within 10 km radius from the farm), and two detection category nodes (use of any PRRS vaccination and type of farm). Detailed information on model parameters is provided in Table 7 and Figure 1 shows the outline of the scenario tree.

**Table 7:** Description of input parameters for individual tree nodes, including their sources and explanations used in the model to assess the pig disease surveillance system through which PRRS is diagnosed in England

**Input parameter (including abbreviations)**	**Value**	**Source and explanation**
Between herd prevalence P*H	0.351	Cross-sectional study
***Risk category nodes***		
Proportion of farms in high pig density area –HDA	0.284	[55]
Proportion of farms using live vaccine- LVAC	*RiskBeta*(53,96)	Cross-sectional study
***Detection category nodes***		
Proportion of farms vaccinating –VAC	*RiskBeta*(64,85)	Cross-sectional study
Proportion of breeding farms –B	0.40	[56]
***Detection nodes:***		
***1. Probability that infected pig shows clinical signs***		
PRRS vaccinated breeding farms – VACB	*RiskBeta*(36,29)	Cross-sectional study
PRRS vaccinated finishing farms-VACF	*RiskBeta*(36,29)	Cross-sectional study
PRRS non-vaccinated breeding farms-NVACB	*RiskBeta*(23,63)	Cross-sectional study
PRRS non-vaccinated finishing farms-NVACF	*RiskBeta*(23,63)	Cross-sectional study
**2. Probability that farmer recognize signs and calls vet**		
PRRS vaccinated breeding farms-VACB	*RiskPert*(0.4,0.5,0.6)	Medium probability [57]: less severe clinical signs were expected due to vaccination which could go unnoticed compare to non-vaccinated farms
PRRS vaccinated finishing farms-VACF	*RiskPert*(0.1,0.2,0.3)	Low probability [57]: it was believed that clinical signs seen in this type of farms are more difficult to recognize compare to breeding farms plus the effect of vaccination resulted in low probability
PRRS non-vaccinated breeding farms-NVACB	*RiskPert*(0.7,0.8,0.9)	High probability [57]: non vaccinated farms are likely to have more naïve population therefore clinical manifestation would be more apparent
PRRS non-vaccinated finishing farms-NVACF	*RiskPert*(0.4,0.5,0.6)	Medium probability [57]: similar to non-vaccinated breeding farms but clinical signs slightly less severe
**3. Probability that vet collects the sample**		
PRRS vaccinated breeding farms-VACB	*RiskPert*(0.4,0.5,0.6)	Medium probability [57]: based on the same assumption as above
PRRS vaccinated finishing farms-VACF	*RiskPert*(0.1,0.2,0.3)	Low probability [57]: based on similar assumption as above
PRRS non-vaccinated breeding farms-NVACB	*RiskPert*(0.7,0.8,0.9)	High probability [57]: based on similar assumption as above
PRRS non-vaccinated finishing farms-NVACF	*RiskPert*(0.4,0.5,0.6)	Medium probability [57]: based on similar assumption as above
**4. Probability that infected animal test positive**		
Sensitivity of PCR test	*RiskUniform*(0.967,0.999)	Evaluation of the AnDiaTec AcuPig PRRSV real time RT-PCR for the detection of NA and EU strains (www.andiatec.com)
Sensitivity of ELISA test	*RiskUniform*(0.961,0.978)	[58]

### Model parameterization

Data from the risk factor analysis were used to model the disease distribution and vaccine use in England. Farmers’ perception as to whether they believed their farm was affected by PRRSV, obtained from the cross-sectional study, was used as an approximation of the proportion of farms with clinical signs suspicious of PRRS. Due to a lack of available information, some assumptions were made when estimating the probability that a farmer would recognize clinical signs and call a veterinarian and the probability that the veterinarian would collect the sample. When making these assumptions, data from the literature on clinical signs awareness [29,59] in combination with expert opinion from a practising specialist pig veterinarian were used.

Defining pig population risk strata was based on 2009 census data of registered pig farms in England [60]. The pig density map [55] was used to estimate the proportion of farms in high density areas. The proportion of farms with live vaccine and the relative risk (RR) for individual risk strata was estimated based on data from the cross-sectional study. For each stratum, the proportion of the reference population and the RR weighted according to the size of stratum population was estimated, giving the average adjusted risk (AR) for the population strata using the formula (Eq.1) described by Martin et al. [54]:

(1) $$\sum_{l}^{L}=\left(AR_{l}\times PrP_{l}\right)=1$$

where L is the number of risk strata and PrP is the proportion of farms in the *l*th stratum.

The effective probability of infection (EPI) for each risk stratum resulted from multiplying the same between herd prevalence (P*H) of PRRSV obtained from the cross-sectional study with the respective AR. Similarly to the risk nodes, the proportion of farms in individual category nodes was estimated using data from the cross-sectional study and agricultural statistics (Table 7). Probability distributions for some of the model parameters were chosen according to the type of data available and are reported as @Risk functions (Table 7).

### Probability of an infected farm being detected

SSC unit sensitivity (CSeU) is the probability that a randomly chosen farm in England will be identified as true positive. This was estimated by summing the positive branches of the scenario tree for individual risk strata [54]. The sum of all infected farms was estimated by summing the branches of the scenario tree classified as infected according to the infection node for each of the risk strata.

The probability that an infected farm would be detected through the passive surveillance system, Pr (ID), was estimated using two equations (Eq.2 and Eq.3).

(1) for the individual risk strata as:

(2) $$\frac{\text{CSeU}\;\text{of}\;a\;\text{risk}\;\text{strata}}{\sum \text{all}\;\text{infected}\;\text{farms}\;\text{in}\;\text{all}\;\text{risk}\;\text{strata}}={\text{Pr}}_{1}\left(\text{ID}\right)$$

(2) for the whole population as:

(3) $$\frac{\sum \text{CSeU}\;\text{of}\;a\;\text{risk}\;\text{strata}}{\sum \text{all}\;\mathrm{infected}\;\text{farms}\;\text{in}\;\text{all}\;\text{risk}\;\text{strata}}={\text{Pr}}_{2}\left(\text{ID}\right)\;$$

Microsoft Excel and Palisade @RISK were used to model variation and uncertainty of model parameters using probability distributions, and the model was simulated with 10,000 iterations.

### Evaluation of input parameters

To assess the impact of variation and uncertainty of model parameters used on model output, a sensitivity analysis was conducted by varying individual input parameters in the model by doubling and halving their values.

## Competing interests

The authors declare that they have no competing interests.

## Authors’ contributions

MV was involved in the design of this study, data collection, carried out statistical analysis and drafted the manuscript. PA participated in data collection and statistical analysis. SW helped with the interpretation and discussion of the results and contributed AHVLA data on clinical signs. BW developed the research concept and idea, designed and coordinated the study, and was involved in the interpretation of the results. PA, SW and BW critically revised the manuscript. All authors read and approved the final manuscript.
